# Fe_3_O_4_@C Nanoparticles Synthesized by In Situ Solid-Phase Method for Removal of Methylene Blue

**DOI:** 10.3390/nano11020330

**Published:** 2021-01-27

**Authors:** Hengli Xiang, Genkuan Ren, Yanjun Zhong, Dehua Xu, Zhiye Zhang, Xinlong Wang, Xiushan Yang

**Affiliations:** 1 School of Chemical Engineering, Sichuan University, Ministry of Education Research Center for Comprehensive Utilization and Clean Processing Engineering of Phosphorus Resources, Chengdu 610065, China; xianghl0908@163.com (H.X.); rgk2000@163.com (G.R.); yjzhong@scu.edu.cn (Y.Z.); dhxu@scu.edu.cn (D.X.); zhiyezhang@scu.edu.cn (Z.Z.); 2 College of Chemistry and Chemical Engineering, Yibin University, Yibin 644000, China

**Keywords:** Fe_3_O_4_@C nanoparticles, solid-phase method, adsorption, Fenton-like reaction, methylene blue

## Abstract

Fe_3_O_4_@C nanoparticles were prepared by an in situ, solid-phase reaction, without any precursor, using FeSO_4_, FeS_2_, and PVP K30 as raw materials. The nanoparticles were utilized to decolorize high concentrations methylene blue (MB). The results indicated that the maximum adsorption capacity of the Fe3O4@C nanoparticles was 18.52 mg/g, and that the adsorption process was exothermic. Additionally, by employing H_2_O_2_ as the initiator of a Fenton-like reaction, the removal efficiency of 100 mg/L MB reached ~99% with Fe_3_O_4_@C nanoparticles, while that of MB was only ~34% using pure Fe_3_O_4_ nanoparticles. The mechanism of H_2_O_2_ activated on the Fe_3_O_4_@C nanoparticles and the possible degradation pathways of MB are discussed. The Fe_3_O_4_@C nanoparticles retained high catalytic activity after five usage cycles. This work describes a facile method for producing Fe_3_O_4_@C nanoparticles with excellent catalytic reactivity, and therefore, represents a promising approach for the industrial production of Fe_3_O_4_@C nanoparticles for the treatment of high concentrations of dyes in wastewater.

## 1. Introduction

Dye pollution is one of the most severe environmental concerns nowadays. Most industrial dyes contain complex components which are highly toxic, teratogenic, and carcinogenic [1,2,3,4]. A host of technologies has been applied for the degradation of dye pollutants including biological, physical, and chemical approaches [5,6,7,8]. Among the various treatments, adsorption and advanced oxidation processes (AOPs) have been shown to be highly efficient methods for the removal of dye from wastewater [9,10]. Adsorption is low-cost and free of intermediates. The Fenton-like system has drawn much attention because of its ability to cleanly and efficiently remove dyes from wastewater. Thus, the development of adsorbents with good performance coupled with Fenton-like reactions has been the focal point of a great deal of recent research.

Because Fe_3_O_4_ has the characteristics of easy magnetic separation, stable properties, and low toxicity, Fe^2+^ and Fe^3+^ can be safely reacted with H_2_O_2_ to trigger the Fenton reaction; in such cases, Fe_3_O_4_ is a potential adsorbent and Fenton catalyst [11,12]. However, the H_2_O_2_-activating ability of pure Fe_3_O_4_ is not strong, and the compound tends to agglomerate in the presence of strong magnetism, which inevitably results in a reduction of the adsorption capacity and catalytic activity [13]. To overcome the shortcomings of single-phase materials [14,15], the design of composite materials has become a focus of today’s research. For example, core-shell structure Fe_3_O_4_/TiO_2_ nanoparticles were successfully manufactured to enhance photocatalytic performance [16,17,18], Glutathione-coated Fe_3_O_4_ was applied in an enhanced photo-Fenton system [19], a novel composite material of g-C_3_N_4_/Fe_2_O_3_/Fe_3_O_4_ was used to degrade Orange II via a visible-light Fenton system [20], hydrothermally synthesized C/Fe_3_O_4_ nanoparticles were used as Fenton-like catalysts with high-performance for dye decolorization [21], a Fe_3_O_4_/WO_3_ core-shell photocatalyst loaded on UiO-66(Zr/Ti) nanoflakes was successfully synthesized to enhance photo-oxidation capacity [22], and a Fe_3_O_4_/CuO@C composite from MOF-based materials was used as a magnetic separation photocatalyst for the degradation of antibiotics [23]. Compared with combinations of Fe_3_O_4_ and metal oxides or polymer shells, the combination with carbon shells has a broader range of practical applications thanks to its stability in acid-base solutions and in high-temperature and high-pressure conditions [24]. Additonally, carbon-based materials are complementary to magnetite because of their conjugated π-electron effect, high porosity, and large specific surface area [25]. Owing to the unique properties of carbon-modified magnetite nanoparticles, such as ease of separation, nontoxicity, and convenient regeneration, these nanoparticles represent a promising method for dye wastewater treatment.

Synthesis methods of carbon-modified magnetite nanoparticles mainly include coprecipitation [26,27,28], the impregnation method [29,30], and hydrothermal carbonization processes [6,15,31], where high production costs and complicated preparation routes hamper industrial production and reduce the feasibility of using these materials to treat dye wastewater on an industrial scale. Hence, the development of a promising method for the preparation of high-performance Fe_3_O_4_@C particles for industrial production is significant. Recently, the synthesisis of nanoparticles by the solid-phase method has drawn much attention because of its low-cost and ease of industrial production. Peng Wang et al. [32] employed the solid-phase method to manufacture Fe_3_O_4_@C nanoparticles using α-Fe_2_O_3_ nanoparticles and acetylene black as raw materials. Kan Wang et al. [33] calcined α-Fe_2_O_3_ nanoparticles in an acetylene atmosphere to obtain γ-Fe_2_O_3_@C nanoparticles, and then calcined the γ-Fe_2_O_3_@C nanoparticles in an N_2_/H_2_ (5% H_2_) atmosphere to acquire Fe_3_O_4_@C nanoparticles. Zhang et al. [34] manufactured porous carbon/Fe_3_O_4_ by the calcination of waste cigarette filters immersed in ferric nitrate solution. However, these solid-phase methods contain a two-step reaction, where the involvement of the solvent and the synthesis of precursors cannot be avoided [35,36], which represents an obstacle to large-scale industrial production. In our previous research, we [37] put forward a new solid-phase reduction process for the synthesis of pure Fe_3_O_4_ nanoparticles with a relatively large particle size, i.e., ~50 nm; however, severe agglomeration caused a low surface area, i.e., 10.6 m^2^/g, which represented one aspect requiring improvement.

In this study, we propose an in situ, solid-phase method to fabricate Fe_3_O_4_@C nanoparticles with core-shell structure using FeSO_4_, FeS_2_, and PVP K30 as raw materials. Characterization of X-ray powder diffraction (XRD), Fourier transform infrared (FT-IR) and Raman spectra, High-resolution transmission electron microscopy (HRTEM), Brunauer-Emmett-Teller (BET) method, Vibrating Sample Magnetometer (VSM), and X-ray photoelectron spectroscopy (XPS) were applied to explore properties of the Fe_3_O_4_@C nanoparticles. The target dye was methylene blue (MB), as it is widely used. The effects of the experimental conditions, adsorption kinetics, and isothermal adsorption were investigated. Furthermore, a Fenton-like reaction was conducted to synergistically degrade high concentration MB, and the synergism between adsorption and the Fenton-like reaction was evaluated. This study presents a facile, in situ, solid-phase method to synthesize Fe_3_O_4_@C nanoparticles for potential industrial-scale production and high-concentration dye wastewater treatment.

## 2. Materials and Methods

### 2.1. Materials

Analytical reagents of FeSO_4_·7H_2_O (99%, Chengdu Kelong Chemical Co, Ltd. Chengdu, China), FeS_2_, (98%, Beijing Hawk Science and Technology Co., Ltd. Bejing, China), PVP K30 ((C_6_H_9_NO)_n_, 99%, Shanghai Yuanye Biotechnology Co., Ltd. Shanghai, China), tert-butanol (99%, Chengdu Kelong Chemical Co, Ltd. Co., Ltd. Chengdu, China), and H_2_O_2_ (30%, Chengdu Kelong Chemical Co, Ltd. Chengdu, China) were used without further purification.

### 2.2. Fabrication of Fe_3_O_4_@C Nanoparticles

The fabrication procedure of the Fe_3_O_4_@C nanoparticles comprised the following steps: (i) FeSO_4_·7H_2_O was dried at 180 °C for 360 min to remove water, yielding FeSO_4_·H_2_O, FeS_2_, and PVP K30, which were also dried at 80 °C until constant weight was achieved. (ii) Then, 15 g FeSO_4_·H_2_O, 1.0 g FeS_2_, and 0.8 g PVP K30 were put into an omnidirectional planetary ball mill and ground for 30 min to obtain a homogeneous mix. (iii) The mixture was placed in a tube furnace at a given heating program (reaction temperature: 500 °C, heating rate: 10 °C/min) under a nitrogen atmosphere. (iv) After calcining, the product was cooled to ambient temperature under nitrogen. (v) The product was removed and washed with deionized water two times, before vacuum drying at 80 °C until constant weight was achieved.

### 2.3. Experimental Procedure for Decolorization of MB

Adsorption:

(1) A certain number of Fe_3_O_4_@C nanoparticles were added to 50 mL MB solutions with various concentrations; these mixtures were then placed on a thermostatic shaker. (2) After starting the thermostatic shaker, the Fe_3_O_4_@C nanoparticles were extracted by a magnet from the heterogeneous solution at selected interval times. (3) A UV-vis spectrophotometer was then used to determine the concentration of methylene blue.

Fenton-like reaction:

(1) A certain number of Fe_3_O_4_@C nanoparticles were added to 50 mL MB solutions with various concentrations; these mixtures were then placed on a thermostatic shaker (2) After running the thermostatic shaker for 60 min to achieve adsorption equilibrium, a certain amount of H_2_O_2_ (30%) was quickly added to the heterogeneous solution to initiate a Fenton-like reaction. (3) Then, 5 mL of the tert-butanol solution (AR) was put into the heterogeneous solution at selected times to inhibit the Fenton-like reaction; the mixture was then centrifuged to remove solid particles. (4) The supernatant liquid obtained from the serum by centrifuge was subjected to a UV-vis spectrophotometer analysis to determine the concentration of methylene blue. Furthermore, pure Fe_3_O_4_ nanoparticles synthesized according to our previous study [37] were used to compare the decolorization efficiency.

### 2.4. Adsorption Kinetic and Interparticle Diffusion Study

The adsorption kinetic models applied in this study were the pseudo-first-order model (Equation (1)), pseudo-second-order model (Equation (2)) [6], and the Elovich model (Equation (3)) [38].

(1) $$\mathrm{In}\;(q_{e}\;-\;q_{t})\;=\;\mathrm{In}\;(q_{e})\;-\;k_{1}t$$

(2) $$\frac{t}{q_{t}}\;=\;\frac{1}{k_{2}q_{e}^{2}}\;+\;\frac{t}{q_{e}}$$

(3) $$q_{t}\;=\;\frac{1}{\beta }\;\mathrm{In}\;(1\;+\;\alpha \beta t)$$

where *q*_t_ (mg/g) is the adsorption capacity at adsorption time t, *k*_1_ (min^−1^) and *k*_2_ (min^−1^) are the rate constants of the pseudo-first-order and pseudo-second-order models, respectively, *α* is the initial adsorption rate (mg/g·min^−1^), and *β* is the desorption constant (g/mg)

The adsorption rate (mg/g min) at the beginning of adsorption was calculated as follows:

(4) $$h\;=\;k_{2}q_{e}^{2}$$

The control-step of the MB adsorption by the Fe_3_O_4_@C nanoparticles was determined by the Weber and Morris model [10]. The expression of the Weber and Morris model may be depicted as followed:

(5) $$q_{t}\;=\;K_{dif}t^{1/2}\;+\;\varepsilon$$

where *K_dif_* (mg/g min^1/2^) is the diffusion rate constant within the adsorption process and *ε* (mg/g) is the dimensionless constant.

### 2.5. Adsorption Isotherm Study

The relevance between the equilibrium adsorption capacity of Fe_3_O_4_@C nanoparticles at different adsorption temperatures and the remaining MB concentration was investigated using adsorption isotherms models. Adsorption isotherm experiments were conducted at 25 °C, 35 °C, and 45 °C. Langmuir, Freundlich, Redlich-Peterson, and Temkin models were introduced to describe the adsorption category [39,40,41]; expressions are shown as Equations (6)–(9).

(6) $$q_{e}\;=\;\frac{q_{\max}k_{LC}C_{equ}}{\left(1\;+\;k_{LC}C_{equ}\right)}$$

(7) qe = KFC Cequ1n

(8) $$q_{e}\;=\;\frac{K_{R}C_{equ}}{\left(1\;+\;a_{R}C_{equ}^{\alpha }\right)}$$

(9) $$q_{e}\;=\;B\;\mathrm{In}({AC}_{equ})$$

where *k_LC_* and *K_FC_* are the Langmuir and Freundlich constant, respectively, *K_R_* and *a_R_* are the characteristics of the R-P isotherm model, *B is* the Temkin constant, A is the equilibrium binding constant, and *C_equ_* is the MB concentration at adsorption equilibrium.

### 2.6. Characteristic Methods

The crystalline structure of the products was verified by XRD (Empyrean, PANalytical, Alemlo, The Netherlands). FT-IR spectroscopy (PerkinElmer Frontier, Waltham, MA, USA) and the Raman spectroscopy (LabRAM HR, Horiba Scientific, Paris, France) were utilized to determine the surface functional radicals of the products. The HRTEM (FEI Talos F200x, Hillsboro, OR, USA) measured the particle morphology, primary particle size, and lattice of the products. The N_2_ adsorption/desorption curves were analyzed by the standard BET method (77 K, NOVA1000e analyzer) to estimate the specific surface area and the corresponding pore structure of the products. The MB solution concentration was measured with a spectrophotometer (664 nm, V-5800, Metash instrument, Shanghai, China), and the intermediates produced under the process of Fenton-like reaction were determined using the LC-MS (Thermo Scientific TSQ Quantum, Waltham, MA, USA). The concentration of leaching iron ions after decolorization was measured using the ICP-AES (DV 7000, Waltham, MA, USA).

## 3. Results

### 3.1. Characterization of the Fe_3_O_4_@C Nanoparticles

**Figure 1:**
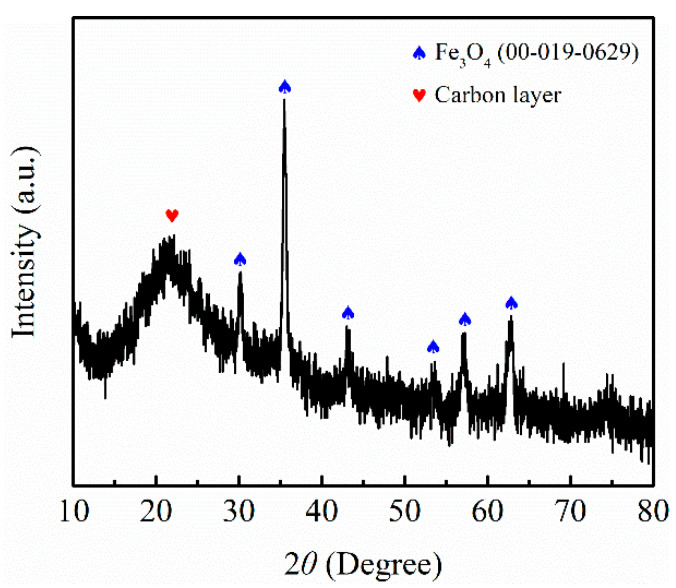
The XRD pattern of the Fe_3_O_4_@C nanoparticles.

The XRD pattern of the Fe_3_O_4_@C nanoparticles shown in Figure 1 indicated that the characteristic peaks of Fe_3_O_4_@C nanoparticles were consistent with the crystal planes of the PDF standard card (JCPDS 00-019-0629) of magnetite [42]. The broad peak found at 2*θ* = 21.6° was the characteristic reflection of carbon [43]. Furthermore, the crystallite sizes measured by Debye-Scherrer’s equation [44] in the light of the strongest diffraction peak (311) was 20.6 nm.

The FT-IR and Raman spectra of the Fe_3_O_4_@C nanoparticles are shown in Figure S1. As shown in Figure S1a, the peaks emerging at 3400 cm^−1^, 1636 cm^−1^, 1123 cm^−1^, and 565 cm^−1^ corresponded to the stretching vibration of the -OH bond [37], the stretching vibration of C=O in amide bond derived from the pyrolysis of the PVP 30 [45], the stretching vibration of the SO_4_^2−^ of the residual ferrous sulfate [46], and the stretching vibration of the Fe^3+^-O [47], respectively. Figure S1b demonstrates that the G band (Graphite) and D band (disordered) of the carbon-carbon bonds could be seen at 1580 cm^−1^ and 1350 cm^−1^, respectively [48,49]. The peak found at 1180 cm^−1^ was the A_1g_ symmetry vibration of the disordered graphitic lattice [50]. Also, the observed diffraction peak at about 670 cm^−1^ was indexed to the A_1g_ mode of magnetite [51]. Therefore, the FT-IR spectroscopy and Raman spectroscopy further demonstrated that Fe_3_O_4_@C nanoparticles had been successfully obtained.

**Figure 2:**
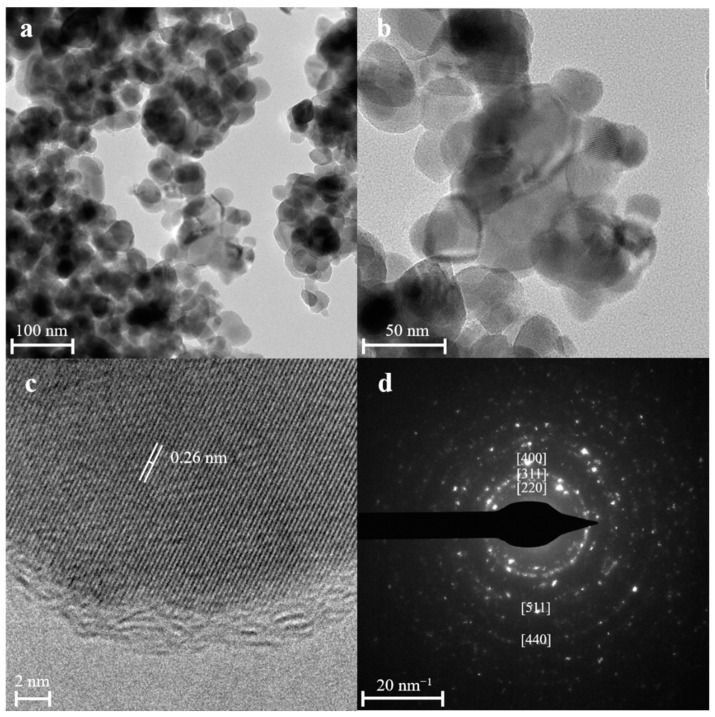
(**a**–**c**) The HRTEM graphs of the Fe_3_O_4_@C, and (**d**) the SAED micrograph of the Fe_3_O_4_@C.

The HRTEM graphs presented in Figure 2a,b show that the morphology of the Fe_3_O_4_@C nanoparticles was spherical, with a core-shell structure. The primary particle diameter was ~30 nm and the thickness of the carbon-shell was ~2 nm. Figure 2c demonstrates that the interlayer spacing of the lattice fringes was 0.26 nm, which closely matched the d-spacing of the (311) plane in cubic Fe_3_O_4_. The SAED micrograph shown in Figure 2d further verified the polycrystalline structure of the Fe_3_O_4_@C nanoparticles, where the diffraction rings could be ascribed to the (220), (311), (400), (511), and (440) planes of Fe_3_O_4_.

The specific surface area of the Fe_3_O_4_@C nanoparticles, calculated by the BET method, according to the N_2_ adsorption/desorption isotherm curves was 37.74 m^2^/g; see Figure S2a. The average pore diameters and pore volume estimated by the BJH method according to the pore distribution, displayed in the inset of Figure S2a, was 3.78 nm and 0.227 cm^3^/g, respectively. The magnetic property of the Fe_3_O_4_@C nanoparticles determined the ease of the separation of the particles in a heterogeneous solution. The magnetic hysteresis loops of the Fe_3_O_4_@C nanoparticles measured by the VSM at 298 K are displayed in Figure S2b, and showed a superparamagnetic feature. The saturation magnetization was 77 emu/g, which was lower than the values reported in the literature [52,53]. The lower saturation magnetization of the as-synthesized Fe_3_O_4_@C nanoparticles might have been due to the coating of the carbon-shell. The coercivity value was found to be only 0.16 kOe and the remnant magnetization was 12.8 emu/g.

### 3.2. Adsorption Studies

**Figure 3:**
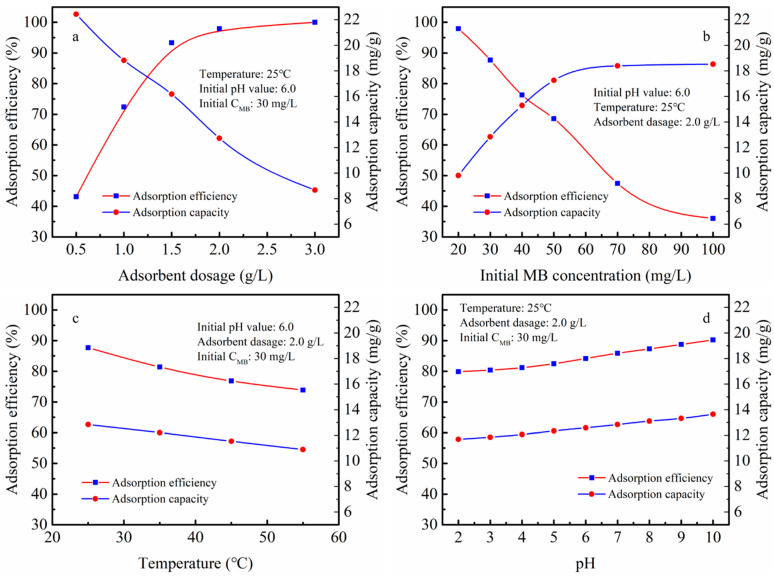
The (**a**) adsorbent dosages, (**b**) initial MB concentrations, (**c**) temperatures, and (**d**) effect of the initial pH values on the adsorption of MB.

The adsorption of MB was the first step of the decolorization of MB. Figure 3 shows the effect of the adsorbent dosages, initial MB concentrations, temperatures, and initial pH values on the adsorption of MB. Figure 3a illustrates that when the adsorbent dosage increased to 2.0 g/L, the adsorption capacity decreased and the adsorption efficiency increased; this was due to the increase of adsorbent dosage that likely increased the adsorption activate site, thereby increasing the adsorption efficiency and decreasing the MB adsorbed per unit mass. Figure 3b shows that as the initial MB concentration increased, the adsorption efficiency gradually declined and the adsorption capacity increased; this was because the addition of the initial MB concentration likely raised the concentration gradient between the MB solution and the Fe_3_O_4_@C nanoparticles. Additionally, the increase of initial MB concentration increased the probability of the MB molecules coming into contact with the active sites on the surface of the adsorbent, so the adsorption capacity for MB improved. Figure 3c implies that the higher the adsorption temperature, the lower the adsorption efficiency, suggesting that the adsorption process was exothermic. Figure 3d reveals that the adsorption capacity and efficiency increased with the initial increase in pH. This was due to the fact that MB is a kind of cationic dye, and a basic solution would reduce the competition between the H^+^ and MB ions, thereby offering more active sites for MB ions [54].

### 3.3. Adsorption Kinetics and Interparticle Diffusion Analysis

**Figure 4:**
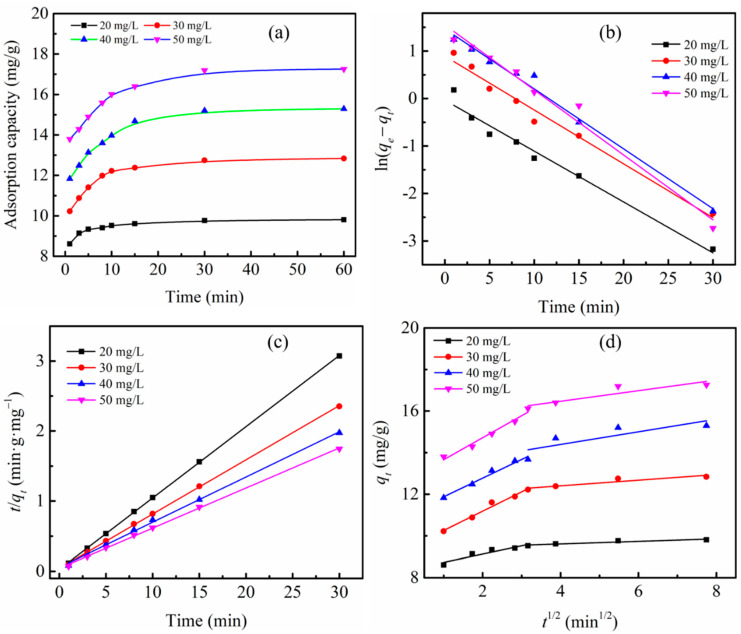
Adsorption kinetics of MB by the as-synthesized Fe_3_O_4_@C nanoparticles: (**a**) effect of adsorption time on adsorption capacity; (**b**) pseudo-first-order; (**c**) pseudo-second-order; (**d**) interparticle diffusion model. (T = 25 °C, initial pH value = 6.0, adsorbent dosage: 2.0 g/L).

The adsorption mechanism of MB by the Fe_3_O_4_@C nanoparticles synthesized by an in situ, solid-phase method is described by adsorption kinetics and interparticle diffusion analyses. It may be seen in Figure 4a that at different initial MB concentrations, the adsorption capacity curve followed the same tendency, and the adsorption capacity increased with the increasing initial C_MB_. Figure 4b,c displays the linear forms of the kinetics model. The correlation coefficients (*R*^2^) and the rate parameters fitted by the kinetic models are given in Table 1. By comparing Figure 4b,c, it is obvious that the pseudo-second-order model better describes the kinetic behavior than the pseudo-first-order model. Additionally, Table 1 also clearly shows that the theoretical adsorption capacity calculated from the pseudo-second-order model provided more accurate results in comparison to the values of the actual adsorption capacity. Furthermore, due to the fact that traditional linear transformation techniques used in adsorption study often misinterpret adsorption processes [55], nonlinear kinetics models were also applied to interpret the adsorption process; the results were shown in Figure S5 and Table S2. As shown in Figure S5 and Table S2, regardless of the linear or nonlinear forms of the kinetics model, the pseudo-second-order model fitted better with the adsorption behavior, while the theoretical adsorption capacity calculated from the pseudo-second-order model was more accurate regarding the values of the actual adsorption capacity. The Weber and Morris model determined the rate-limiting steps of the adsorption process. Figure 4d shows a graph of *q_t_* versus *t*^1/2^, while the parameters of the interparticle diffusion model of MB adsorption are illustrated in Table 2. The results of the plots shown in Figure 4d presented multilinearity and the intercept was not 0; as such, the adsorption process of MB on Fe_3_O_4_@C nanoparticles included interparticle diffusion and boundary layer diffusion [6]. Additionally, the heterogeneous diffusion process controlled by the reaction rate and diffusion factor, including a series of reaction mechanisms such as the diffusion of solutes at the liquid phase or interface, surface activation, and deactivation, was described by the Elovich model; the results are shown in Figure S6 and Table S3. The *α* values of the Elovich model were much higher than the *β* values, indicating that the adsorption rate was much higher than the desorption rate [38]. In addition, the *R*^2^ values of the Elovich model were higher than those of both the pseudo-first- and pseudo-second-order models, suggesting that the Elovich model best represents the experimental kinetic data.

### 3.4. Adsorption Isotherm Study

**Figure 5:**
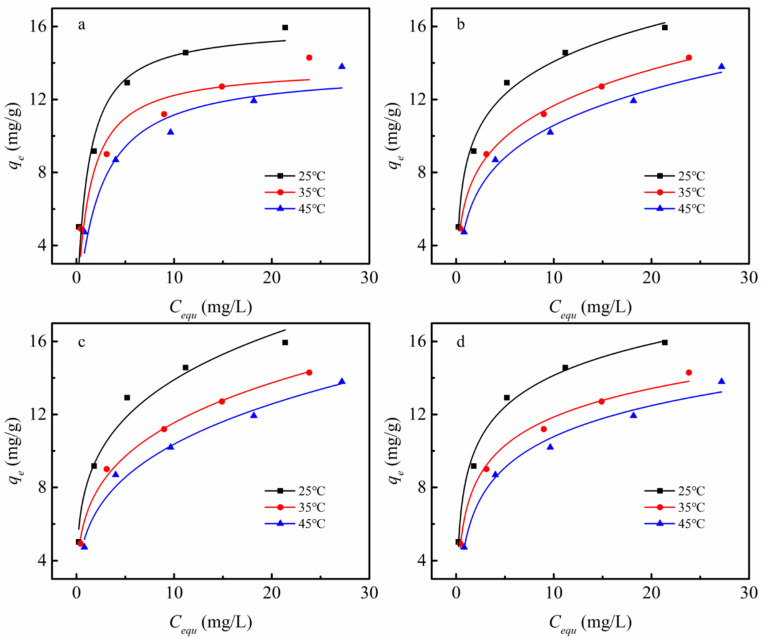
Adsorption isotherms of (**a**) Langmuir model, (**b**) Freundlich model, (**c**) Redlich-Peterson model, and (**d**) Temkin model for methylene blue adsorption by Fe_3_O_4_@C nanoparticles. (Time: 1 h, initial pH: 7.0, the adsorbent dosage of 2.0 g/L).

An adsorption isotherm study was used to analyze the relationship between the equilibrium adsorption capacity of the Fe_3_O_4_@C nanoparticles and the remaining concentration of the MB solution at a selected temperature [56]. Figure 5 and Table 3 display the Langmuir, Freundlich, Redlich-Peterson, and Temkin models and the fitting parameters of the MB adsorption by the Fe_3_O_4_@C nanoparticles. It may be seen in Figure 5 and Table 3 that the *R*^2^ of the Langmuir model was lower than those of the Freundlich, Redlich-Peterson, and Temkin models, indicating that the adsorption of MB on the Fe_3_O_4_@C nanoparticles was not a single-layer adsorption on a uniform surface [3]. The Freundlich model was based on multilayer adsorption on a reversible heterogeneous surface, considering that the Fe_3_O_4_@C nanoparticles tend to agglomerate because of their magnetic characteristics. As such, the Freundlich model may be more appropriate for describing the adsorption behaviour [4]. However, the Redlich-Peterson model showed a higher value of *R*^2^ compared to the Freundlich model, which implied that the adsorption behaviour of MB on the Fe_3_O_4_@C nanoparticles possessed a hybrid characteristic of the traditional Langmuir and Freundlich models. Therefore, the Redlich-Peterson model could be used to describe the relationship between the equilibrium adsorption capacity of the Fe_3_O_4_@C nanoparticles and the remaining concentration of the MB solution under a selected temperature. The Temkin model considered the effects of the indirect adsorbate/adsorbent interactions on the distribution of adsorption heat and binding energies. The *R*^2^ values were all above 0.98, which further demonstrated that the adsorption active sites on the Fe_3_O_4_@C nanoparticle surfaces were not uniform, and the increase of temperature likely reduced the binding ability between the MB molecules and the Fe_3_O_4_@C nanoparticles.

### 3.5. Fenton-Like Reaction

**Figure 6:**
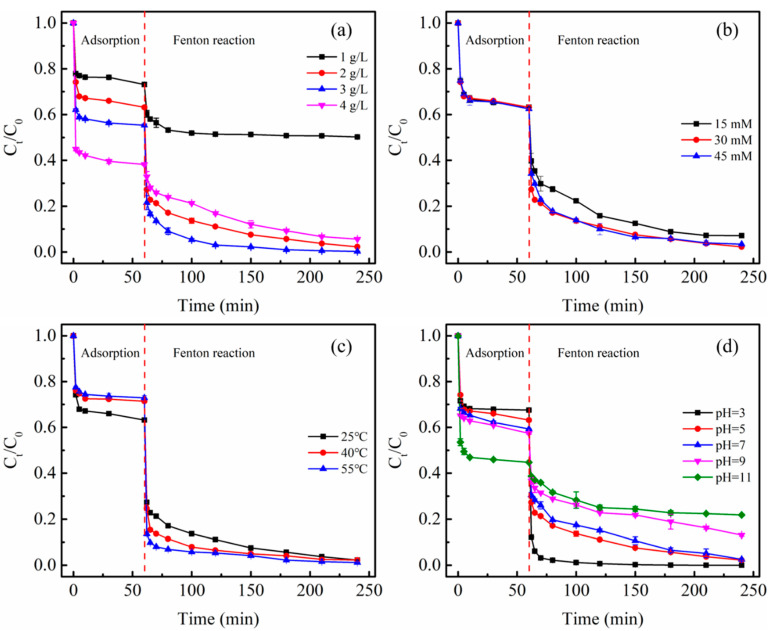
(**a**) Adsorbent dosage, (**b**) H_2_O_2_ concentration, (**c**) temperature, and (**d**) initial pH affections on the decolorization efficiency of MB. Except for the studied parameters, other parameters were fixed at pH = 5.0, Fe_3_O_4_@C = 2.0 g/L, [H_2_O_2_] = 30 mM, [MB] = 100 mg/L, and temperature 25 °C.

By only carrying out adsorption, the adsorption efficiency of Fe_3_O_4_@C nanoparticles on MB of 100 mg/L was lower than 40%. In this case, H_2_O_2_ was used as an additive to activate a Fenton-like reaction in order to achieve synergetic degradation of a high concentration of MB. The influence of the operational parameters on MB decolorization is displayed in Figure 6. Figure 6a depicts the effect of Fe_3_O_4_@C nanoparticle dosage on MB decolorization by Fenton-like reaction. With an increase of Fe_3_O_4_@C nanoparticle dosage from 1.0 g/L to 3.0 g/L, the decolorization efficiency rose from ~50% to ~99%. However, with a further increase to 4.0 g/L, the decolorization efficiency decreased; this result was caused by the excessive iron species, which led to competitive scavenging of radicals with MB molecules, thereby decreasing the decolorization efficiency [57]. Figure 6b shows that as the concentration of H_2_O_2_ increased from 15 mM to 30 mM, the decolorization rate and efficiency increased significantly, and when the concentration of H_2_O_2_ reached 45 mM, the decolorization rate and efficiency exhibited no difference compared with 30 mM of H_2_O_2_. Normally, more H_2_O_2_ led to a higher decolorization efficiency, but excess H_2_O_2_ also resulted in a reaction with •OH to form HO_2_•, which delayed MB decolorization [58]. From Figure 6c, it may be seen that although higher temperatures were not favorable to the adsorption reaction, for the Fenton reaction, the decolorization rate and efficiency were higher at high temperatures. The initial pH value was an important parameter for the Fenton-like reaction, as this could strongly influence the amount of •OH radicals produced by the leaching Fe^2+^ and H_2_O_2_. As shown in Figure 6d, the higher pH values resulted in higher adsorption efficiency, while lower pH values were more favorable for the leaching of iron ions, which accounted for higher decolorization efficiency and rate.

In this study, the concentration of MB solution was much higher than that in most of the literature, and the dosage of the catalysts and H_2_O_2_ was not excessive. A comparison of different catalysts for MB degradation through the Fenton-like reaction is shown in Table 4.

### 3.6. The Mechanism of the Decolorization of MB

**Figure 7:**
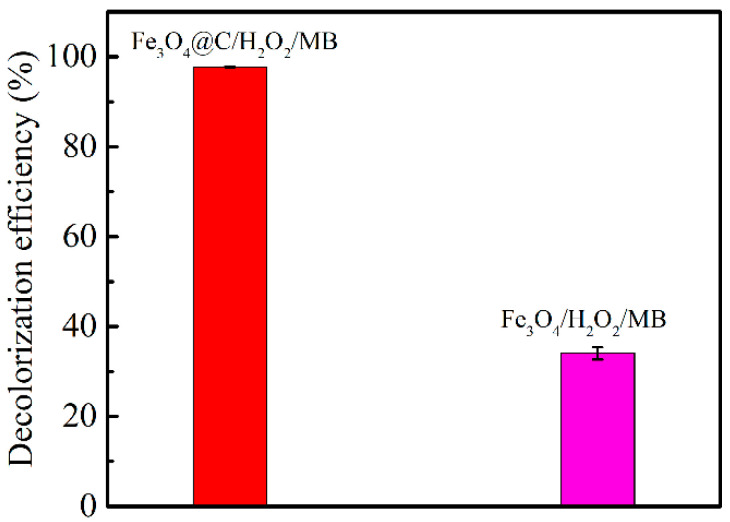
Comparison of Fenton-like catalytic activity between Fe_3_O_4_@C nanoparticles and pure Fe_3_O_4_ nanoparticles.

A comparison of the Fenton-like catalytic activity between Fe_3_O_4_@C nanoparticles and pure Fe_3_O_4_ nanoparticles was made for the removal of 100 mg/L MB for 180 min with a Fe_3_O_4_@C dosage of 2.0 g/L, an initial pH value of 3.0, and a temperature of 25 °C; see Figure 7. According to Figure 7, the pure Fe_3_O_4_ nanoparticles exhibited much lower decolorization of MB (~34%) than the Fe_3_O_4_@C nanoparticles (~99%).

**Figure 8:**
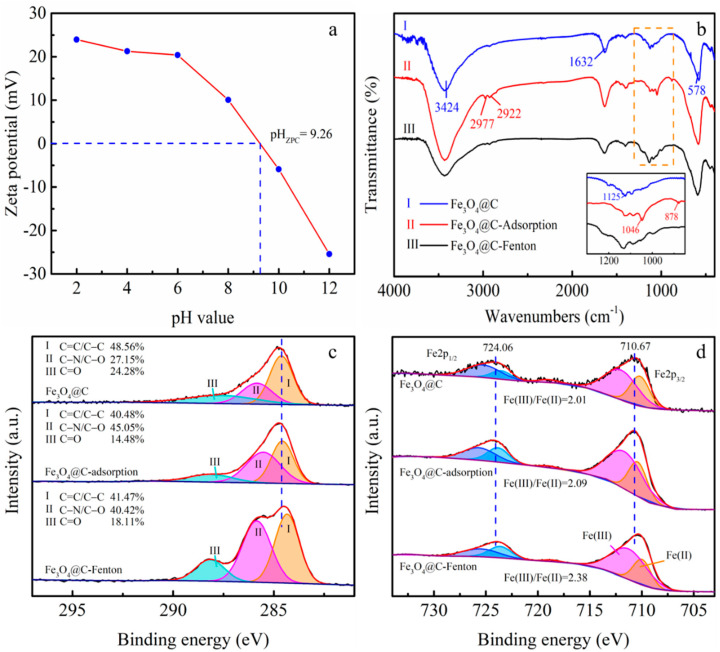
(**a**) The Zeta potential of the Fe_3_O_4_@C nanoparticles, the (**b**) FT-IR spectra, (**c**) C1s spectra, and (**d**) Fe2p spectra of the Fe_3_O_4_@C nanoparticles, Fe_3_O_4_@C nanoparticles after adsorption, and Fe_3_O_4_@C nanoparticles after Fenton-like reaction.

This remarkable difference in decolorization efficiency was because the carbon-shell could adsorb more MB molecules and H_2_O_2_ molecules to produce more radicals (•OH), thereby accelerating the removal of the MB in the solution. Based on the results of the Raman spectrum and FT-IR spectrum, the carbon-shell on the Fe_3_O_4_ nanoparticles was mostly amorphous (I_D_/I_G_ = 1.2) and the main bonds on the surface were C=O and C-N. The carbon-shell, rich in the functional groups, could easily adsorb the MB molecules and H_2_O_2_ molecules to initiate the Fenton-like reaction on the particle surface. Additionally, the carbon-shell also inhibited particle growth, thereby increasing the specific surface area and providing more adsorption active sites. The Zeta potential of the Fe_3_O_4_@C nanoparticles, displayed in Figure 8a, suggested that the pH_ZPC_ of the Fe_3_O_4_@C nanoparticles was 9.26, which meant that the surface of Fe_3_O_4_@C nanoparticles was negatively charged. As MB is a cationic dye with a positive charge, the electrostatic attraction and conjugated π-electron effect were the primary mechanisms for the adsorption of MB on the Fe_3_O_4_@C nanoparticles.

The FT-IR spectra of Fe_3_O_4_@C nanoparticles, Fe_3_O_4_@C nanoparticles after adsorption of MB, and Fe_3_O_4_@C nanoparticles after Fenton-like reaction, as shown in Figure 8b, further confirmed this hypothesis. Figure 8b shows that after adsorption of MB, new peaks emerged at 878 cm^−1^ and 1046 cm^−1^, which were assigned to the C-H in the benzene ring and the rocking vibration of -CH_3_, while the peaks at 2977 cm^−1^ and 2922 cm^−1^ were ascribed to the stretching vibration of -CH_3_, indicating that the MB had been adsorbed by the nanoparticles [69]. However, after the Fenton-like reaction was complete, the FT-IR spectra showed no difference compared to Fe_3_O_4_@C nanoparticles, indicating that the MB had been successfully degraded. In addition, XPS was used to investigate the valence changes of the Fe_3_O_4_@C nanoparticles before and after adsorption and Fenton-like reaction. As displayed in Figure 8c, the peak of C1s could be deconvoluted to the peaks of C-C/C=C, C-N/C-O, and C=O [70]. The ratio of C-N/C-O after adsorption of MB increased from 27.15% to 45.05%, suggesting that the MB molecules had been successfully adsorbed on the surface of the Fe_3_O_4_@C nanoparticles. However, when the Fenton-like reaction was complete, the ratio of C-N/C-O decreased from 45.05% to 40.42% and the ratio of C=O increased from 14.48% to 18.11%, suggesting that part of the carbon-shell had been oxidized during the Fenton-like reaction [63]. Furthermore, the peak of C-C/C=C of the Fe_3_O_4_@C nanoparticles at the binding energy of 284.60 eV shifted to 284.30 eV, which was caused by the oxidation of unstable amorphous carbon and the exposure of graphite. The peaks of Fe2p, as shown in Figure 8d, indicated that the Fenton-like reaction triggered on the surface of nanoparticles resulted in a change of the ratio of Fe(III)/Fe(II) from 2.01 to 2.38, because a small amount of Fe(II) was oxidized to Fe(III).

**Figure 9:**
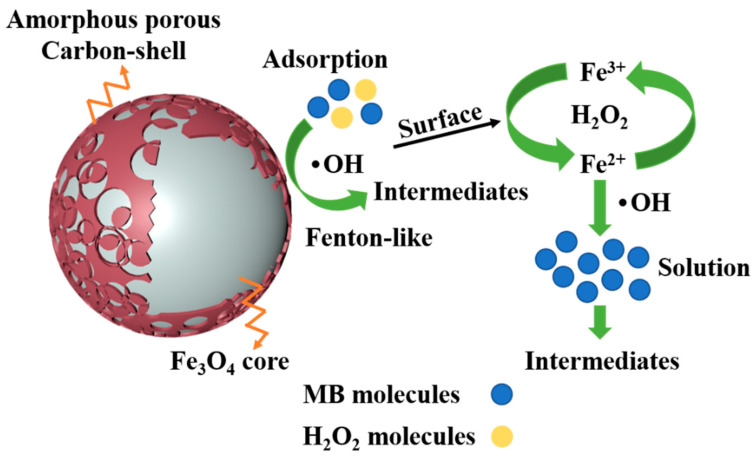
The activation mechanism of H_2_O_2_ on Fe_3_O_4_@C nanoparticles.

The proposed mechanism of degradation of MB is shown in Figure 9. Firstly, the Fe_3_O_4_@C nanoparticles could easily adsorb MB and H_2_O_2_ molecules due to electrostatic attraction and the conjugated π-electron effect. Then, the H_2_O_2_ molecules could react with the Fe ions to generate •OH to degrade MB molecules. The excessive •OH would diffuse into the solution to degrade more MB, and the Fe_3_O_4_@C nanoparticles would continuously adsorb MB molecules for synergetic degradation. Thus, by employing the Fenton-like reaction to promote adsorption, the degradation ability for high concentration MB was significantly enhanced.

### 3.7. Possible Degradation Pathways of MB

**Scheme 1:**
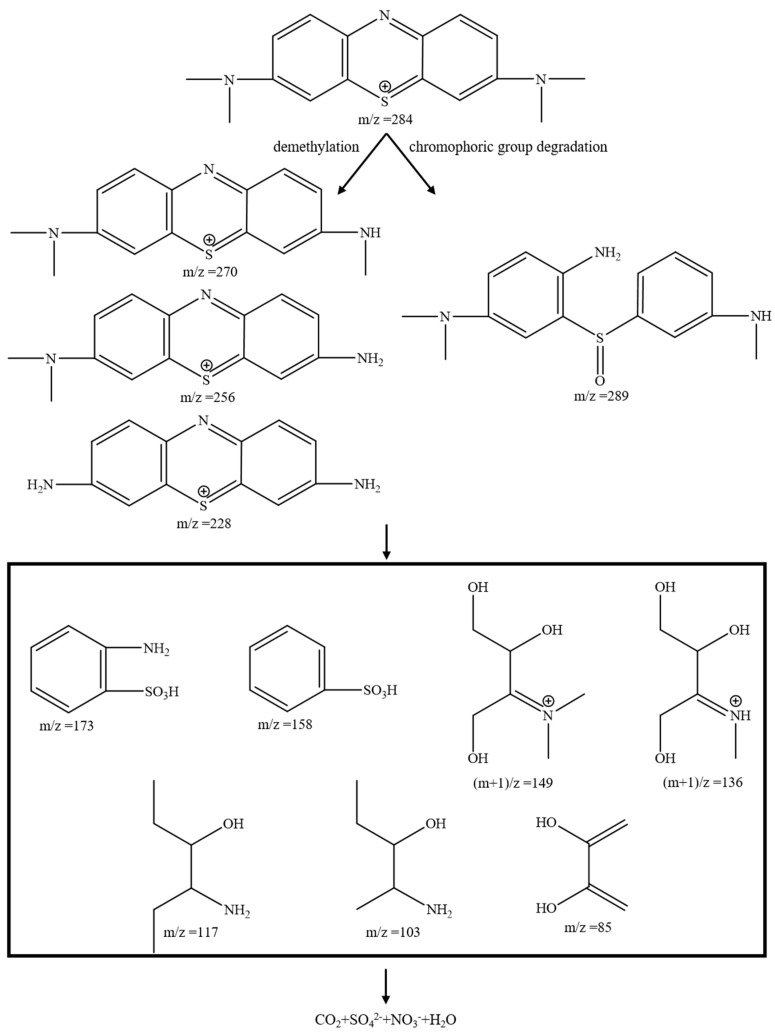
The possible degradation pathway of MB.

The possible degradation pathways of MB were determined by LC-MS analysis; the ESI mass spectra results at reaction times of 1 h and 3 h are presented in Figures S3 and S4, and the possible intermediate degradation products of MB are presented in Table S1. From Figure S3, it may be seen that at a reaction time of 1 h, the initial reaction step was the demethylation of MB to form Azure A (m/z = 270), B (m/z = 256), and C (m/z = 241). Meanwhile, the degradation of the chromophoric group was detected where the electronic reorganization led to a change of C-S^+^=C to C-(S=O)-C and the break of C-N-C to form C-NH_2_ [71,72]. Then, under the attack of •OH, the aromatic groups were continuously destroyed, giving rise to smaller intermediate products at m/z = 173, 158, 149, 136, 117, 103, and 85 [73,74,75]. Figure S4 indicates that the main structure of the MB molecule was destroyed at a reaction time of 3 h, and only smaller intermediate products were detected by the ESI mass spectra, indicating that the MB molecule had been successfully degraded. The possible degradation pathways of MB are displayed in Scheme 1. In addition, after 3 h of the MB removal (conditions: 100 mM MB, 30 mM H_2_O_2_, 2 g/L Fe_3_O_4_@C nanoparticles, 40 °C, and initial pH value of 3.0), the removal efficiency of TOC reached 82.38%, where the remaining TOC in water was 14.90 mg/L, indicating that most of the methylene blue molecules had been completely mineralized, and that those that had not had at least been decomposed into small molecular intermediates.

### 3.8. The Recyclability Tests of the Fe_3_O_4_@C Nanoparticles

**Figure 10:**
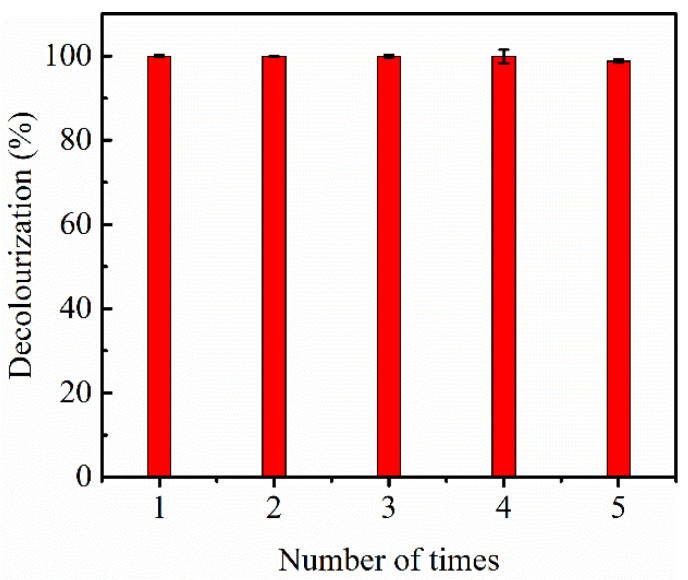
The decolorization efficiency of the Fe_3_O_4_@C nanoparticles. (Conditions: 100 mM MB, 30 mM H_2_O_2_, 2 g/L Fe_3_O_4_@C nanoparticles, the temperature of 40 °C, and initial pH value of 3.0).

Recycling experiments were implemented to examine the stability of the Fe_3_O_4_@C nanoparticles. As shown in Figure 10, the decolorization ratio remained ~97% after five cycles of use under the optimum conditions, which indicated excellent stability. Furthermore, the leaching of iron ions, as determined by ICP-OES, was 1.14 mg/L after the decolorization process, suggesting that ions leached from Fe_3_O_4_@C nanoparticles would not give rise to secondary pollution. The stability of the carbon-shell with respect to reaction with •OH radicals was determined using a carbon-sulfur analyzer. The results shown in Figure S7 suggested that the carbon contents in the Fe_3_O_4_@C nanoparticles reduced from 1.47 wt.% to 1.17 wt.% after five usage cycles, indicating the relatively high stability of the carbon-shell. Figure S8 also demonstrates that the reduction of the carbon contents in the Fe_3_O_4_@C nanoparticles resulted in a reduction of the adsorption efficiency from 32.5% to 24.1% after five usage cycles; however, after employing the Fenton-like reaction, the final decolorization of the MB remained ~97% after five usage cycles, indicating excellent stability of the Fe_3_O_4_@C nanoparticles.

## 4. Conclusions

Fe_3_O_4_@C nanoparticles were fabricated by an in situ, solid-phase method without any precursors, and were employed for the decolorization of MB. Characterization showed that the Fe_3_O_4_@C nanoparticles had been successfully prepared with a primary particle size of ~30 nm and a carbon-shell with a thickness of ~2 nm. The XPS and FT-IR spectra demonstrated that the carbon-shell mainly comprised C=O and C-N bonds. The specific surface area, average pore diameters, and pore volume of the Fe_3_O_4_@C nanoparticles was 37.74 m^2^/g, 3.78 nm, and 0.227 cm^3^/g, respectively. The saturation magnetization, coercivity, and remnant magnetization of the Fe_3_O_4_@C nanoparticles was 77 emu/g, 0.16 kOe, and 12.8 emu/g, respectively. The as Fe_3_O_4_@C nanoparticles showed a much lower average particle size and much higher specific surface area compared to pure Fe_3_O_4_ nanoparticles synthesized by the solid-phase method, demonstrating that the adjunction of PVP K30 in the solid-phase method significantly improved the particle properties. Moreover, it was shown that the Fe_3_O_4_@C/H_2_O_2_ system could effectively decolorize MB through the simultaneous involvement of the adsorption and Fenton-like process, where the carbon-shell provided adsorption active sites for MB and H_2_O_2_ molecules, while the core Fe_3_O_4_ provided Fe ions to stimulate the Fenton-like reaction. The maximum adsorption capacity of Fe_3_O_4_@C nanoparticles for MB was 18.52 mg/g, and the adsorption kinetic was well-fitted by the Elovich model, indicating that the adsorption process was a heterogeneous diffusion process. Additionally, the Redlich-Peterson adsorption isotherm model could better describe the adsorption behavior, implying that the adsorption active sites on the surface of the Fe3O4@C nanoparticles were not uniform, and that the increase of temperature reduced the binding ability between the MB molecules and the Fe_3_O_4_@C nanoparticles. Also, to degrade higher concentrations of methylene blue solution, H_2_O_2_ was added after the adsorption equilibrium to stimulate the Fenton reaction. The removal efficiency of 100 mg/L MB reached ~99% by Fe_3_O_4_@C nanoparticles after 3 h, and the maximum decolorization of the MB was still more than 97% after five usage cycles. Compared to the efficiency of different catalysts for MB degradation described in the literature, the Fe_3_O_4_@C nanoparticles proposed in this paper could degrade MB of much higher concentration without excessive usage of catalyst or H_2_O_2_. The leaching iron ions in the solution, as determined by ICP-OES, constituted 1.14 mg/L, suggesting that the Fe_3_O_4_@C nanoparticles had good stability. The possible degradation pathways of the MB molecule were determined by LC-MS: demethylation, chromophoric group crack, and aromatic ring opening to form smaller fragments. The primary limitations of this study are that the raw materials to synthesize Fe_3_O_4_@C nanoparticles were all analytical, pure reagents, and that application in real dye wastewater has not been studied. In future research, we will focus on the use of titanium dioxide waste residue (~90% FeSO_4_·7H_2_O) and pyrite (~75% FeS_2_) to synthesize the Fe_3_O_4_@C nanoparticles by the in situ, solid-phase method, and its application in real dye wastewater. In conclusion, this study describes a promising method for the industrial production of Fe_3_O_4_@C nanoparticles and their potential for industrial treatment of high concentration dye wastewater.

## Figures and Tables

**Table 1:** Kinetic parameters for adsorption of methyl blue on Fe_3_O_4_@C nanoparticles.

*C_ini_* g·L^−1^	*q_e(expe)_* mg/g	Pseudo-First-Order Kinetics			Pseudo-Second-Order Kinetics			
		*k_1_* min^−1^	*q_e(calc)_* mg/g	$R_{1}^{2}$	*k_2_* g/mg∙min	*q_e(calc)_* mg/g	$R_{2}^{2}$	*h* mg/mg∙min
20	9.809	0.1018	0.9962	0.9704	0.3924	9.827	0.9992	37.89
30	12.84	0.1133	2.445	0.9795	0.1464	12.93	0.9997	24.47
40	15.29	0.1262	4.333	0.9862	0.0794	15.51	0.9990	19.10
50	17.26	0.1369	4.718	0.9784	0.0775	17.48	0.9991	23.68

**Table 2:** The parameters of the interparticle diffusion model of methyl blue adsorption.

*C_int_* mg/L^−1^	*K_dif, 1_* mg/g·min^0.5^	*ɛ_dif, 1_* mg/g	$R_{dif,\;1}^{2}$	*K_dif, 2_* mg/g·min^0.5^	*ɛ_dif, 2_* mg/g	$R_{dif,\;2}^{2}$
20	0.3964	8.336	0.8735	0.0610	9.369	0.8126
30	0.9254	9.336	0.9735	0.1353	11.86	0.8196
40	0.8963	10.98	0.9699	0.3039	13.17	0.6355
50	1.0562	12.60	0.9671	0.2554	15.45	0.7411

**Table 3:** Parameters of the Langmuir and Freundlich adsorption isotherm models for MB adsorbed on Fe_3_O_4_@C nanoparticles.

***Temp*** **.** **°C**	**Langmuir**			**Freundlich**		
	***q*** **_max_** **mg/g**	***k_LC_*** **L/mg**	$R_{L}^{2}$	***K_FC_*** **g/mg∙min**	**1/*n***	$R_{FC}^{2}$
25	16.00	0.8955	0.8954	8.060	0.2365	0.9643
35	14.19	0.7804	0.8711	6.486	0.2503	0.9939
45	13.69	0.4383	0.9021	5.468	0.2779	0.9800
***Temp*** **.** **°C**	**Redlich-Peterson**			**Temkin**		
	***a_R_*** **(L/mg)^α^**	***K_R_*** **L/mg**	$R_{R}^{2}$	***B***	***A*** **L/mg**	$R_{T}^{2}$
25	5.963	58.11	0.9830	2.488	29.36	0.9873
35	5.496	43.11	0.9971	2.450	19.26	0.9874
45	3.762	25.03	0.9878	2.254	8.159	0.9804

**Table 4:** The comparison of different catalysts for MB degradation through the Fenton-like reaction.

Catalysts	[Catalysts] (g/L)	[H_2_O_2_] (mM)	[MB] (mg/L)	Removal Efficiency	Ref.
MnMg/Fe LDH	1.0	10	20	93% in 300 min	[59]
Fe_3_O_4_/CuO	1.6	32	10	95% in 120 min	[60]
MPCMSs	2.0	16	40	~100% in 25 min	[61]
Fe_3_O_4_/rGO	0.3	60	10	~99% in 120 min	[62]
Fe_3_O_4_/C	0.5	90	10	~100% in 60 min	[63]
Fe_3_O_4_/rGO aerogel	0.3	20	50	~100% in 360 min	[64]
Fe_2_O_3_	0.5	30	10	~70% in 420 min	[65]
MIL-68(Fe)	0.2	50	20	~100% in 40 min	[66]
CuCr_2_O_4_/CeO_2_	1.0	4	10	~80% in 20 min	[67]
CuFe_2_O_4_/Cu@C	0.5	16	20	~100% in 30 min	[68]
Fe_3_O_4_@C	2.0	30	100	~99% in 180 min	This study

## Data Availability

The data presented in this study are available on request from the corresponding author.

## Supplementary Materials

The following are available online at https://www.mdpi.com/2079-4991/11/2/330/s1, Figure S1: The (**a**) FT-IR spectroscopy and (**b**) Raman spectroscopy of the as-synthesized Fe_3_O_4_@C nanoparticles, Figure S2: (**a**) The N_2_ adsorption/desorption isotherm curves and (**b**) the magnetic property of the Fe_3_O_4_@C nanoparticles, Figure S3: The non-linear forms of the kinetics model. (**a**) pseudo-first-order model, (**b**) pseudo-second-order model, Figure S4: The Elovich kinetics model of the adsorption, Figure S5: ESI mass spectra of different retention time at the reaction time of 1 h, Figure S6: ESI mass spectra of different retention time at the reaction time of 3 h, Figure S7: The carbon contents of the Fe_3_O_4_@C nanoparticles after repeated use, Figure S8: The recyclabitily test of the Fe_3_O_4_@C nanoparticles (Conditions: 100 mM MB, 30 mM H_2_O_2_, 2 g/L Fe_3_O_4_@C nanoparticles, the temperature of 40℃, and initial pH value of 3.0), Table S1: Kinetic parameters for adsorption of methyl blue on Fe_3_O_4_@C nanoparticles, Table S2: The Elovich kinetic parameters for adsorption of methyl blue on Fe_3_O_4_@C nanoparticles, Table S3: The possible intermediate degradation products of MB.
