# Supplementary material for: Fe3O4@C Nanoparticles Synthesized by In Situ Solid-Phase Method for Removal of Methylene Blue
Source: Nanomaterials (Basel). 2021 Jan 27;11(2):330. doi: 10.3390/nano11020330 (PMC7912336; doi:10.3390/nano11020330)
Supplement: Supplementary file 1 [file nanomaterials-11-00330-s001.pdf]

nanomaterials

MDPI

Supplementary Materials

Fe_3O_4@C Nanoparticles Synthesized by In Situ Solid-Phase Method for Removal of Methylene Blue

Hengli Xiang [1], Genkuan Ren [1,2], Yanjun Zhong [1], Dehua Xu [1], Zhiye Zhang [1], Xinlong Wang [1,*] and Xiushan Yang [1,*]

1 School of Chemical Engineering, Sichuan University, Ministry of Education Research Center for Comprehensive Utilization and Clean Processing Engineering of Phosphorus Resources, Chengdu 610065, China; xianghl0908@163.com (H.X.); rgk2000@163.com (G.R.); yjzhong@scu.edu.cn (Y.Z.); dhxu@scu.edu.cn (D.X.); zhiyezhang@scu.edu.cn (Z.Z.)
2 College of Chemistry and Chemical Engineering, Yibin University, Yibin 644000, China
* Correspondence: wangxl@scu.edu.cn (X.W.); yangxs@scu.edu.cn (X.Y.)

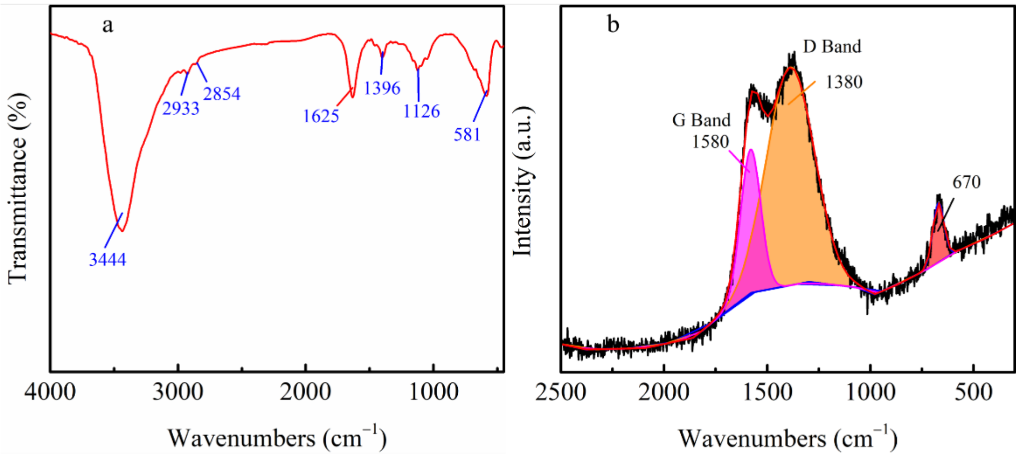

Figure S1. The (**a**) FT-IR spectroscopy and (**b**) Raman spectroscopy of the as-synthesized Fe_3O_4@C nanoparticles.

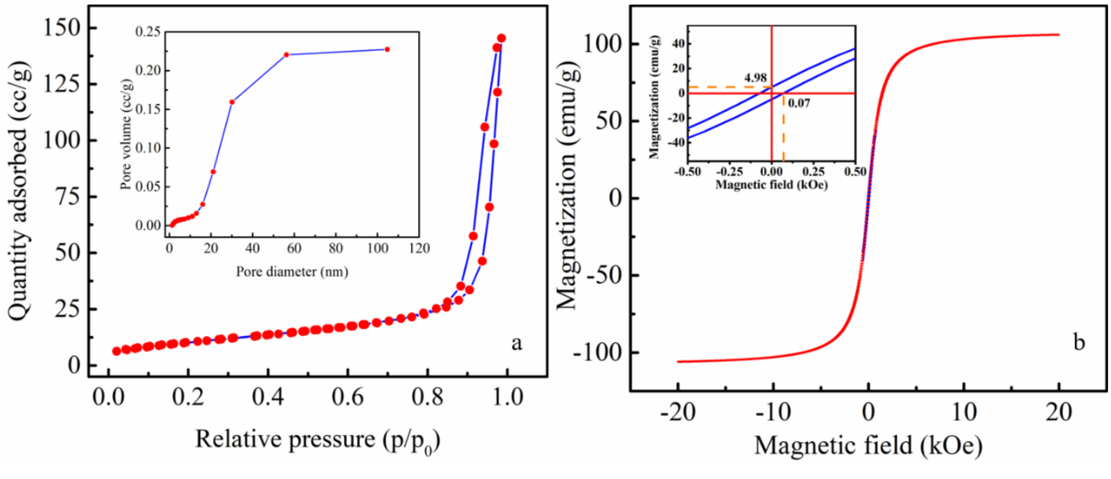

Figure S2. (**a**) The N_2 adsorption/desorption isotherm curves and (**b**) the magnetic property of the Fe_3O_4@C nanoparticles.

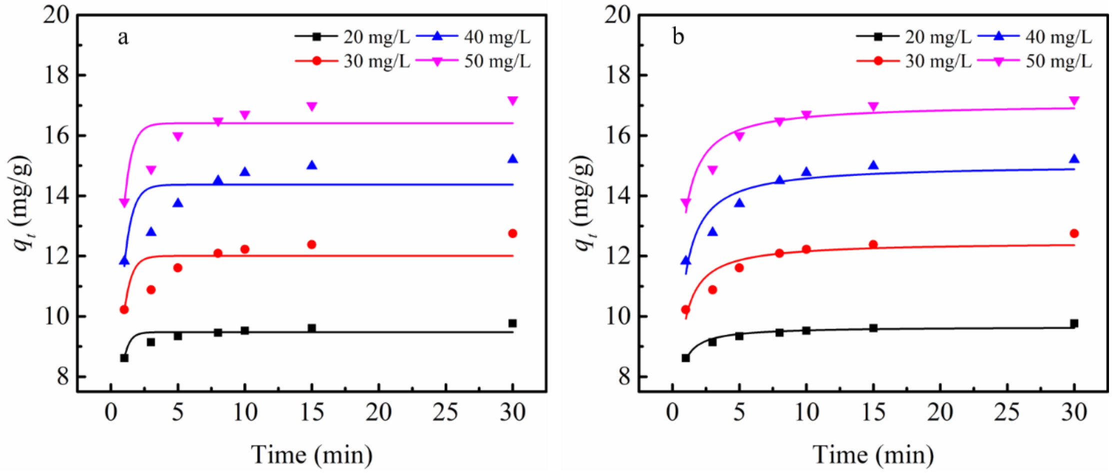

Figure S3. The non-linear forms of the kinetics model. (**a**) pseudo-first-order model, (**b**) pseudo-second-order model.

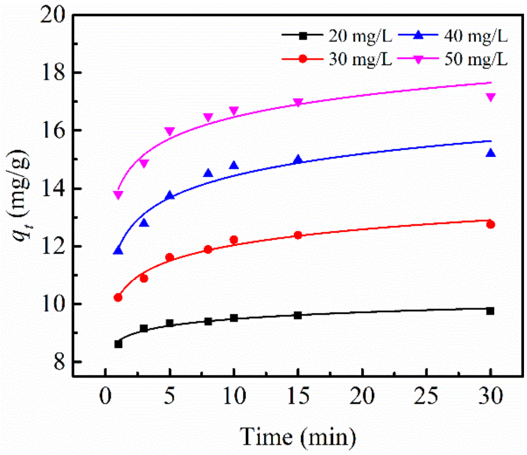

Figure S4. The Elovich kinetics model of the adsorption.

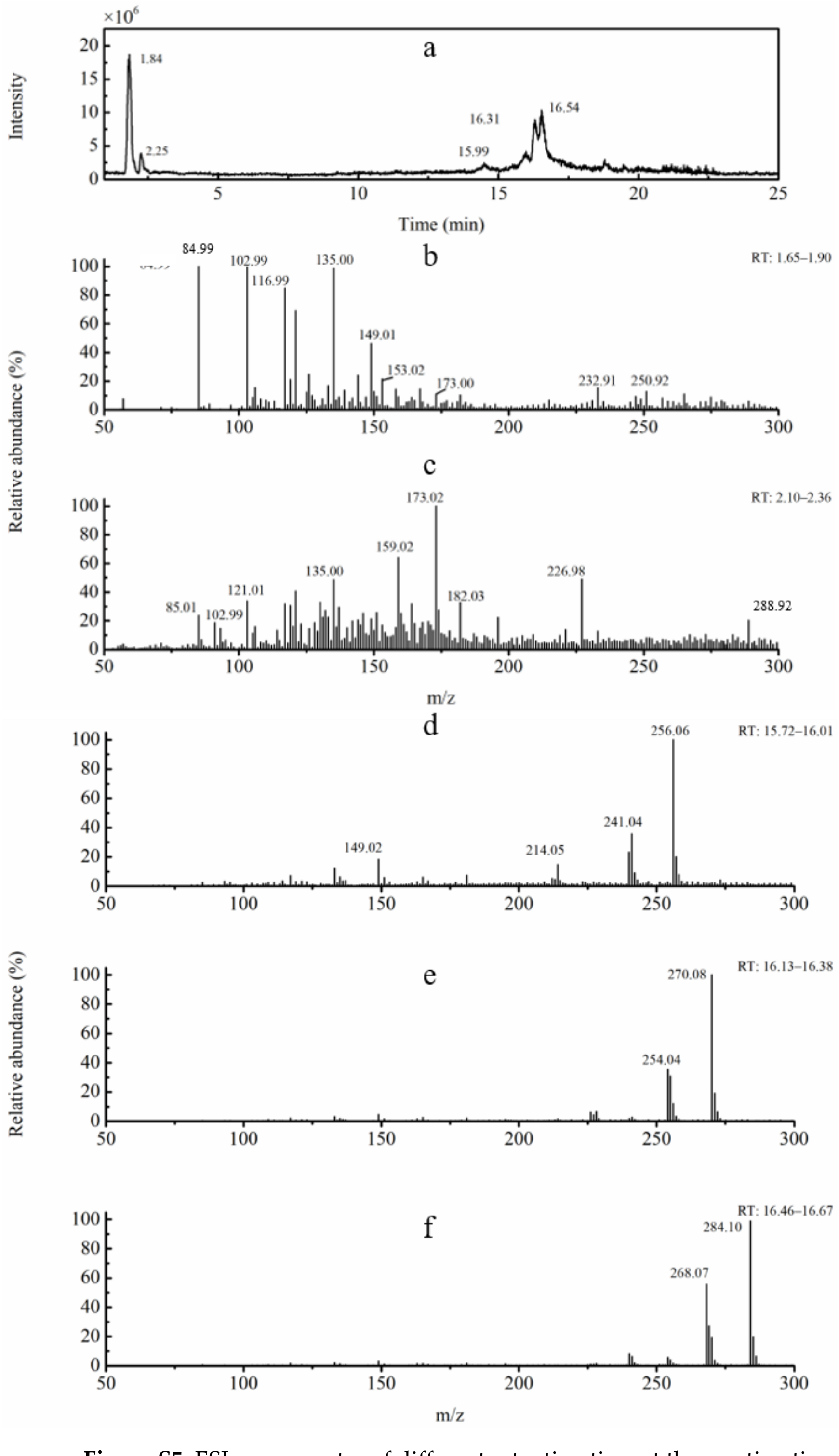

Figure S5. ESI mass spectra of different retention time at the reaction time of 1 h.

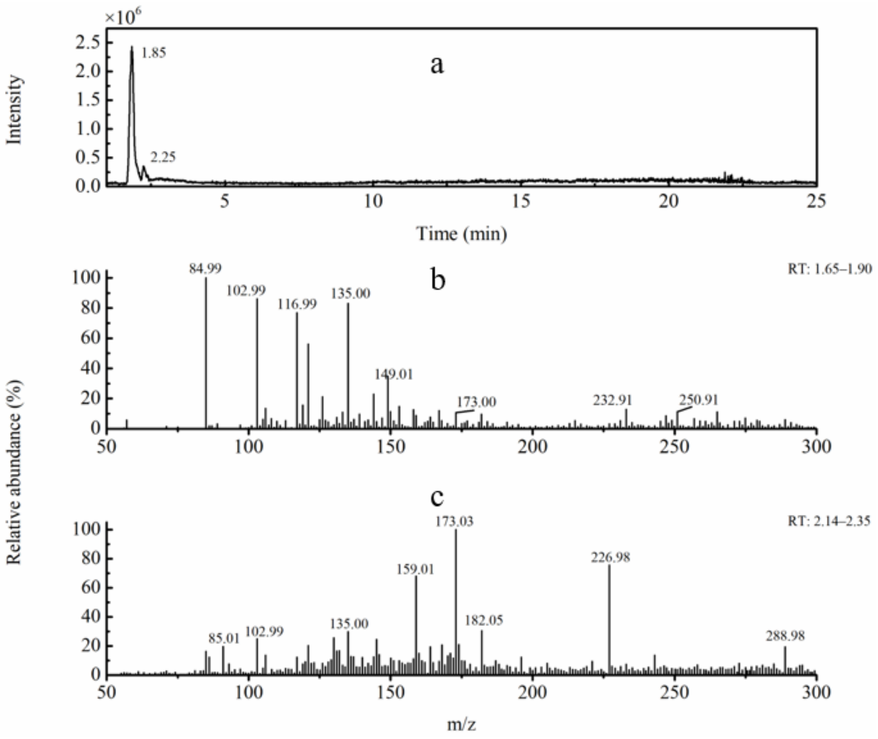

Figure S6. ESI mass spectra of different retention time at the reaction time of 3 h.

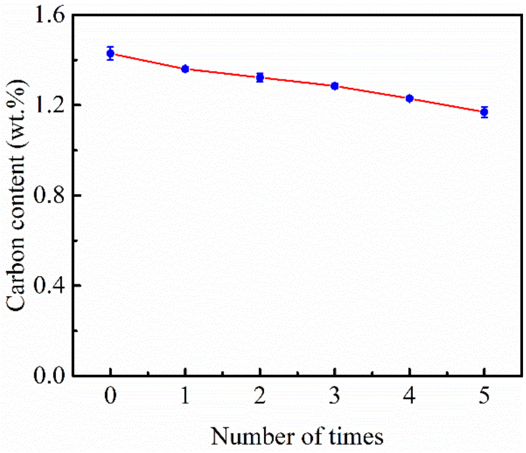

Figure S7. The carbon contents of the Fe_3O_4@C nanoparticles after repeated use.

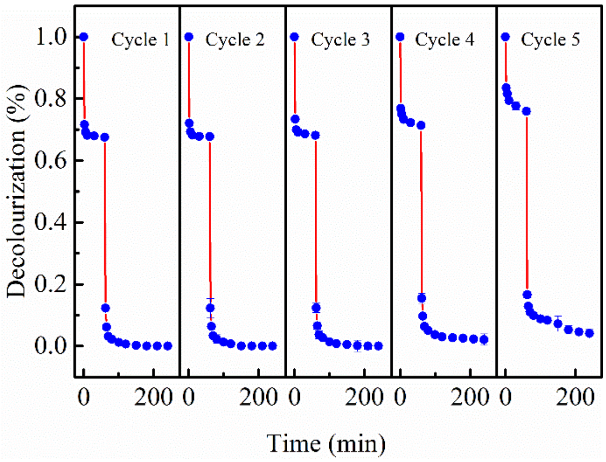

Figure S8. The recyclabitily test of the Fe_3O_4@C nanoparticles (Conditions: 100 mM MB, 30 mM H_2O_2, 2 g/L Fe_3O_4@C nanoparticles, the temperature of 40℃, and initial pH value of 3.0).

Table S1. Kinetic parameters for adsorption of methyl blue on Fe_3O_4@C nanoparticles.

C_{ini} mg × L^{-1}	$q_{e(expe)}$ mg/g	Pseudo-First-Order Kinetics			Pseudo-Second-Order Kinetics			
		k_1 min^{-1}	$q_{e(calc)}$ mg/g	R_1^2	k_2 g/mg·× min	$q_{e(calc)}$ mg/g	R_2^2	h mg/mg·× min
20	9.809	2.384	9.477	0.6822	0.5706	9.659	0.9326	53.23
30	12.84	1.863	12.01	0.4845	0.1651	12.95	0.8290	27.69
40	15.29	1.792	14.37	0.5084	0.1117	15.32	0.8470	26.22
50	17.26	1.672	16.41	0.5679	0.0918	17.38	0.8832	27.73

Table S2. The Elovich kinetic parameters for adsorption of methyl blue on Fe_3O_4@C nanoparticles.

C_{ini} mg·× L^{-1}	Elovich Model		
	α mg/(g × min)	β g/mg	R_1^2
20	6.928×10^{10}	2.984	0.9577
30	3.339×10^{5}	1.269	0.9706
40	5.569×10^{4}	0.924	0.9325
50	4.380×10^{4}	0.910	0.9296

Table S3. The possible intermediate degradation products of MB.

No.	m/z	Structural Formula
1	289	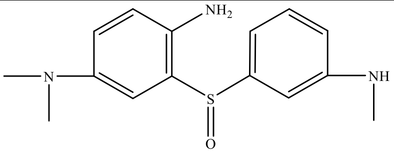
2	284	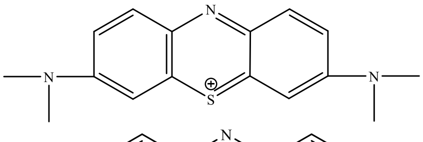
3	270	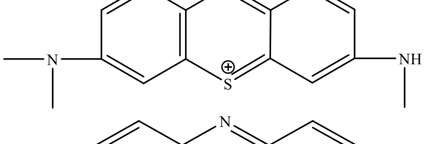
4	256	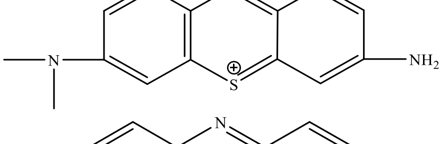
5	227	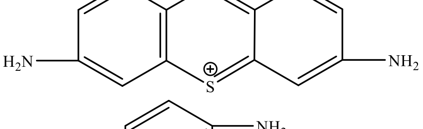
6	173	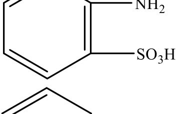
7	159	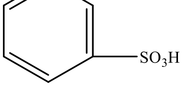

8	149	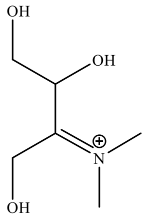
9	135	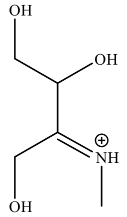
10	117	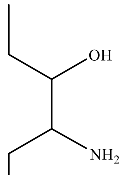
11	103	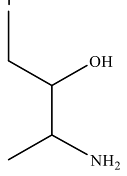
12	85	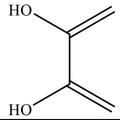
